# Supplementary material for: Content-rich biological network constructed by mining PubMed abstracts
Source: BMC Bioinformatics. 2004 Oct 8;5:147. doi: 10.1186/1471-2105-5-147 (PMC528731; doi:10.1186/1471-2105-5-147)
Supplement: Additional File 2 — The original results of the above study (non-essential files are deleted to keep the file size under the limit set by BMC bioinformatics). [file 1471-2105-5-147-S2.bz2 › chilibotAdditionalFile2/dip05/90ID7962001E327/html/RECO_SSB.html]

 


 **RECO** and **SSB** 
  
Found 13 abstracts in PubMed, retrieved 05.  
 

 What does Google say? 
 PDF only 
| .edu only 

---

**Interactive relationship** (e.g. stimulation, inhibition, etc)

**Inhibitory relationship**- RecR protein, which is known to stimulate  **RecO**  protein to facilitate  **SSB**  protein displacement by RecA protein, inhibits annealing by  **RecO**  protein, suggesting that RecR protein may regulate the choice between the DNA strand invasion versus annealing pathways.  Ref: 12438681 Proc Natl Acad Sci U S A, 2002
**Neutral relationship**- Optimal activity for  **RecO**  protein occurs after formation of a 1 1 complex with  **SSB**  protein.  Ref: 12438681 Proc Natl Acad Sci U S A, 2002
